# Subclinical corneal inflammation and subbasal nerve alterations in keratoconus detected by in vivo confocal microscopy: a cross-sectional study

**DOI:** 10.1007/s00417-024-06664-x

**Published:** 2024-11-09

**Authors:** Chareenun Chirapapaisan, Methawee Sawarot, Sathiya Kengpunpanich

**Affiliations:** Department of Ophthalmology, Faculty of Medicine Siriraj Hospital, Mahidol University, 2 Wanglang Road, Siriraj, 10700 Bangkok Thailand

**Keywords:** Subclinical corneal inflammation, Subbasal nerves, Keratoconus, In vivo confocal microscopy (IVCM)

## Abstract

**Purpose:**

To investigate the intracorneal inflammation and subbasal nerve alterations in keratoconus.

**Methods:**

This prospective cross-sectional study recruited patients with keratoconus, who were diagnosed and graded the severity based on clinical examination and Schiempflug tomography. Laser in vivo confocal microscopy (IVCM) was performed on the corneal subbasal layer centrally to explore the inflammatory cells (ICs), subbasal nerve density (SND), and nerve tortuosity. Keratoconus severity and related factors including ocular allergy, systemic atopy, eye rubbing, floppy eyelids, and contact lens use were recorded. Association between the factors, IC density, SND and keratoconus severity were analyzed.

**Results:**

Thirty-four keratoconus eyes were enrolled, and their IVCM findings were compared with those of 20 age-matched normal eyes. Keratoconus showed a significant increase in ICs (44.25 ± 7.01 vs. 13.06 ± 7.51 cells/mm^2^, *p* < 0.001) and a significant decrease in SND (16.54 ± 0.79 vs. 20.62 ± 0.72 mm/mm^2^, *p* < 0.001) when compared to controls. The alterations were pronounced in severe keratoconus as the IC density was significantly higher (*p* < 0.001), whereas SND was lower (*p* = 0.001) in high-graded keratoconus than in low-graded keratoconus. However, there was no significant correlation between the number of IC and SND in keratoconus eyes (*p* = 0.835). Corneal sensitivity and nerve tortuosity were not different between keratoconus and the controls. No keratoconus-related factors were associated with IC density except the severity of keratoconus (*p* < 0.001, 95% CI [0.70, 0.95]).

**Conclusion:**

Keratoconus, a clinically noninflammatory corneal disease, demonstrates subclinical corneal inflammation and subbasal nerve decline as shown by IVCM. These alterations correlate considerably with the severity of keratoconus.

**Key messages:**

***What is known***

- Traditionally, Keratoconus is a clinically noninflammatory corneal disease.

***What is new***

- Our study suspected keratoconus may be subclinical corneal inflammatory disease.
- In our research, A Keratoconus patient was discovered to have corneal inflammation and a reduction in sub-basal nerve density through the use of In Vivo Confocal Microscopy.
- Increase in corneal inflammation is considerably correlated with the severity of keratoconus.

## Background

Keratoconus (KC) is the most common corneal ectatic disorder [1]. It is characterized by progressive thinning at the central or paracentral area of the cornea, resulting in a cone-shaped protrusion and corneal deformity.

The current diagnosis of KC is based on clinical manifestations and corneal tomographic data. The clinical diagnosis consists of multiple changes of manifest refraction, which range from progressive myopia and astigmatism and the presence of the clinical characteristics of KC on slit-lamp biomicroscopic examination (Fleischer rings, Vogt’s striae, Charleaux oil droplet, Munson’s sign and Rizzuti’s sign) [1, 2]. Regarding the corneal tomographic diagnosis, key composite findings are steepening of the corneal curvature, abnormally high elevation of the anterior and posterior corneal surfaces and localized corneal thinning corresponding to the area showing an abnormally high curve or elevation [3]. The pathogenesis of KC has been presumed to have 3 possible elements [4–6]: 1) an epithelial abnormality that causes the release of proteolytic enzymes that degrade corneal stromal collagen; 2) a chronic microtrauma to Bowman’s layer, resulting in breaks or fragments of Bowman’s layer and stromal thinning; 3) minor changes in the cellular pleomorphism of the endothelium secondary to contact lens use, induce corneal hypoxia, or mechanical stress. However, the exact etiology of KC is not well established. The literature reveals that environmental factors such as ocular allergies, floppy eyelid syndrome, and eye rubbing have been strongly associated with KC [4, 7]. A genetic predisposition in some specific races [8] and systemic syndromes (e.g. Down syndrome, Ehlers-Danlos syndrome, Turner syndrome and Marfan syndrome) have also been reported as being associated with KC [9]. Among these factors, ocular allergies and eye rubbing were significantly found in most of the patients with KC [10–13].

In the past decade, researchers have investigated the association between ocular allergies and the progression of keratoconus [14–17]. Ahuja et al. discovered an increase in immunoglobulin E (IgE) levels in the serum of patients with KC [14]. They demonstrated that the tear film of patients with KC had increased levels of pro-inflammatory cytokines e.g. interleukin 6 (IL-6), tumor necrosis factor-alpha (TNF-α), and matrix metalloproteinase-9,13 (MMP-9,13) [16–18]. Repetitive mechanical trauma to the corneal epithelium by rubbing the eye may cause an imbalance of inflammatory molecules and the recruitment of responsive inflammatory cells (ICs) into the superficial cornea [18]. Although KC is generally considered a noninflammatory disease, there is evidence that dormant subtle inflammation all over the patient’s body or around the eye somehow provokes corneal structural changes, that turn a normal cornea into keratoconus or worsen an existing keratoconus eye [16, 19].

In vivo confocal microscopy (IVCM) has been widely used to analyze corneal and ocular surface diseases [20]. This optical imaging technique facilitates the real-time, noninvasive visualization of intracorneal structures and pathology. Detecting morphological changes and ICs within the cornea becomes feasible [20–23].

To date, no data have yet validated intracorneal inflammation in KC. The current study aimed to investigate ICs in the corneas of patients with various KC grades using IVCM. Detecting a significant rise in corneal ICs may elucidate the hidden pathogenesis of keratoconus or its aggravating factors, which would benefit research on treating KC. We also analyzed the subbasal nerves of patients with KC, as alterations to the nerves could support the existence of prolonged corneal inflammation.

## Methods

This prospective cross-sectional study was conducted in compliance with the ethical principles of the Declaration of Helsinki and was approved by the Ethics Committee of the Siriraj Institutional Review Board, Faculty of Medicine Siriraj Hospital, Mahidol University, Thailand. Participants were informed about the study protocol and gave written consent before recruitment. All study procedures were performed at the Ophthalmology Department, Siriraj Hospital, Bangkok, Thailand.

### Study population

Patients were recruited if they were diagnosed with KC by a cornea specialist (C.C.). The diagnosis was based on clinical manifestations and corneal tomography, per the Global Consensus on Keratoconus and Ectatic Disease 2015 [24]. The corneal tomography was obtained with a Schiempflug tomographer (Pentacam HR, Oculus Optikgerate GmbH, Wetzlar, Germany). The severity of KC was classified as stage 1 to 4 using the ABCD Keratoconus Grading System [25]. We further divided the keratoconic eyes into two subgroups: low-grade KC (stages 1–2) and high-grade KC (stages 3 and 4). After performing corneal tomography, associated factors were elicited and recorded. The factors were age, sex, family history of KC, systemic atopy, ocular allergies, history of chronic eye rubbing, floppy eyelids, contact lens use, and anti-allergy medication use. Patients with contact lenses were asked to refrain from using the lenses for at least one month before returning to participate in the study and repeat the corneal tomography to obtain a definite KC grading. Corneal sensation was assessed with a Cochet-Bonnet esthesiometer (Luneau Ophthalmologie, Chartres, France) per the standard technique [26]. The entire ocular surface, including the eyelids, was examined thoroughly. Dry eye disease was also evaluated with the Oxford grading schema using fluorescein staining. Afterward, patients were sent for corneal confocal microscopy.

Patients were excluded from this study if any of the following applied: 1) they demonstrated advanced KC (grade 4 with a history of corneal hydrops); 2) they had substantial corneal scarring at the central cornea; 3) dry eye disease exceeding grade 2 of the Oxford scheme for grading corneal and conjunctival staining [27], with clinical ocular surface inflammation; 4) previous ocular surgery, a history of ocular trauma or diseases other than KC. Healthy adult subjects were recruited into the study as age-matched normal controls. They were aged between 20 and 50 years and drawn from the Siriraj Health-Screening Center. While either one or two eyes from each patient were enrolled if they were eligible, only one eye from each normal subject was randomly selected.

### In vivo confocal microscopy (IVCM)

**Fig. 1:**
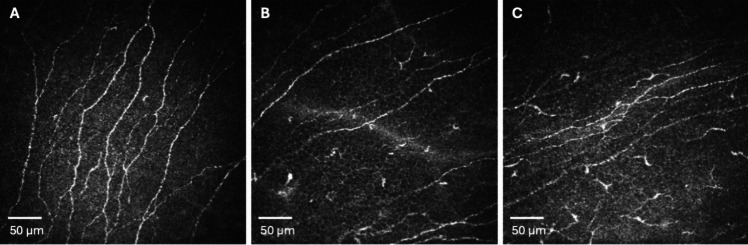
In vivo confocal images demonstrate inflammatory cells (IC) and subbasal nerves in the normal eye compared with those in keratoconus. **A** A normal eye shows high nerve density with scarce ICs, **B** low-grade KC has a slight decrease in nerve density with some ICs, **C** high-grade KC has relatively lower nerve density and a greater increase in ICs compared to low-grade KC

Laser IVCM (Heidelberg Retina Tomograph 3 with the Rostock Cornea Module [HRT3/RCM]; Heidelberg Engineering GmbH, Germany) was performed bilaterally in the central cornea of all subjects. The sequence mode function of the IVCM device was employed to scan the corneal subbasal layer per a technique previously described [28]. Multiple IVCM scans were randomly performed to collect images of ICs dwelling in the basal epithelial and subbasal layers. The three best representative confocal images of ICs and subbasal nerves from each eye were selected. They were analyzed by two masked observers (C.C. and M.S.). The confocal images demonstrating ICs and subbasal nerves in the normal and KC eye were shown in Fig. 1.

### Analysis of inflammatory cell density

Corneal dendritic cells (DCs) are easily recognized by their distinct characteristics of a bright cellular body and dendrites [29]. As they account for the vast majority of ICs induced during corneal inflammation, it is taken for granted that detected DCs can represent ICs. In the current study, the ICs in the basal epithelial and subbasal layers were identified and manually quantified by two masked observers (C.C. and M.S.), using ImageJ software (National Institutes of Health, Bethesda, Maryland). The IC densities of the patients with KC were compared with those derived from age-matched healthy controls.

### Subbasal nerve analysis

Analysis and quantification of the corneal subbasal nerves were undertaken. To this end, two masked observers (C.C. and M.S.) used the semiautomated, nerve-tracing program NeuronJ, version 1.4.3 (a plug-in for ImageJ: http://rsb.info.nih.gov/ij/; http://www.imagescience.org/meijering/software/neuronj) [30]. The nerve parameters and tortuosity of the patients with KC and the normal controls were compared. The nerve parameters included the number and density of the main nerve trunks, nerve branches, and total nerves. The subbasal nerve tortuosity was clinically graded into five categories (0–4), per the classification of Oliviera-Soto et al. [31]

### Outcome measures

The primary outcome was the density of ICs detected in the central cornea of patients with KC, compared with the density of the normal controls. The secondary outcomes were the alterations to the corneal subbasal nerves of the patients with KC and the correlation between the number of ICs, subbasal nerve density, and KC-related factors (sex, family history of KC, systemic atopy, ocular allergies, eye rubbing, floppy eyelids, contact lens use, anti-allergy medication use, and KC severity).

### Statistical analysis

The analyses were performed with IBM SPSS statistics for Windows, version 26.0 (IBM Corp, Armonk, NY, USA). The baseline data of the patients and the controls were reported as the means and standard deviations, and the patient and control group data were compared using the chi-squared test and Student’s t-test. Generalized estimating equations (GEE) were applied to compare the IC densities and subbasal nerve parameters of the keratoconic eyes and the controls, accounting for within-subject variables. The correlations between IC density, subbasal nerve density, and KC-related factors were also assessed by GEE. Probability (p) values less than 0.05 were considered statistically significant.

Based on the number of cases analyzed in previous studies related to KC-associated factors [32, 33] and our method of sample size calculation, which was predicated on an anticipated 20% increase in IC density from normal, indicative of inflammation, using a 90% power and a 95% confidence level, the estimated number of eyes required for a valid analysis was 34 for the study group (KC) and 17 for the normal controls.

## Results

Thirty-four eyes of 20 KC patients were eligible for this study, while 20 age-matched healthy eyes served as controls. The demographic data of the patients with keratoconic and normal eyes are presented in Table 1. Fourteen patients had high-grade KC, while four patients had both high- and low-grade KC in either eye, and another four patients had low-grade KC. The majority of eyes, accounting for 27 eyes (79.41%), presented with high-grade KC, while 7 eyes (20.59%) were classified as low-grade KC.

**Table 1:** Demographic data of patients with keratoconus (KC) and controls

	KC	Controls	*P*-value
Number (eyes)	34	20	-
Age (years)	26.14 ± 7.56	27 ± 2.6	0.507
Sex, male (%)	22 (64.71%)	14 (57.14%)	0.750
Study eye, right eye (%)	18 (51.43%)	8 (40%)	0.548
BCVA (logMAR)	0.27 ± 0.41	0	0.001*
High-graded keratoconus (eyes, %)	27 (79.41%)	-	-
Corneal sensation (cm)	4.83 ± 2.02	6	0.264

BCVA = best-corrected visual acuity, represented as Logarithm of Minimum Angle of Resolution (LogMAR)

All data are presented with mean ± standard deviation

*Statistical significance when *p* < 0.05 compared between study groups

The best-corrected visual acuity (BCVA) was significantly worse in the keratoconic eyes (LogMAR 0.27 ± 0.41 vs. 0.00; *p* < 0.001). Otherwise, there were no significant differences in the KC and control groups’ age, sex, eye laterality, or corneal sensation.

**Fig. 2:**
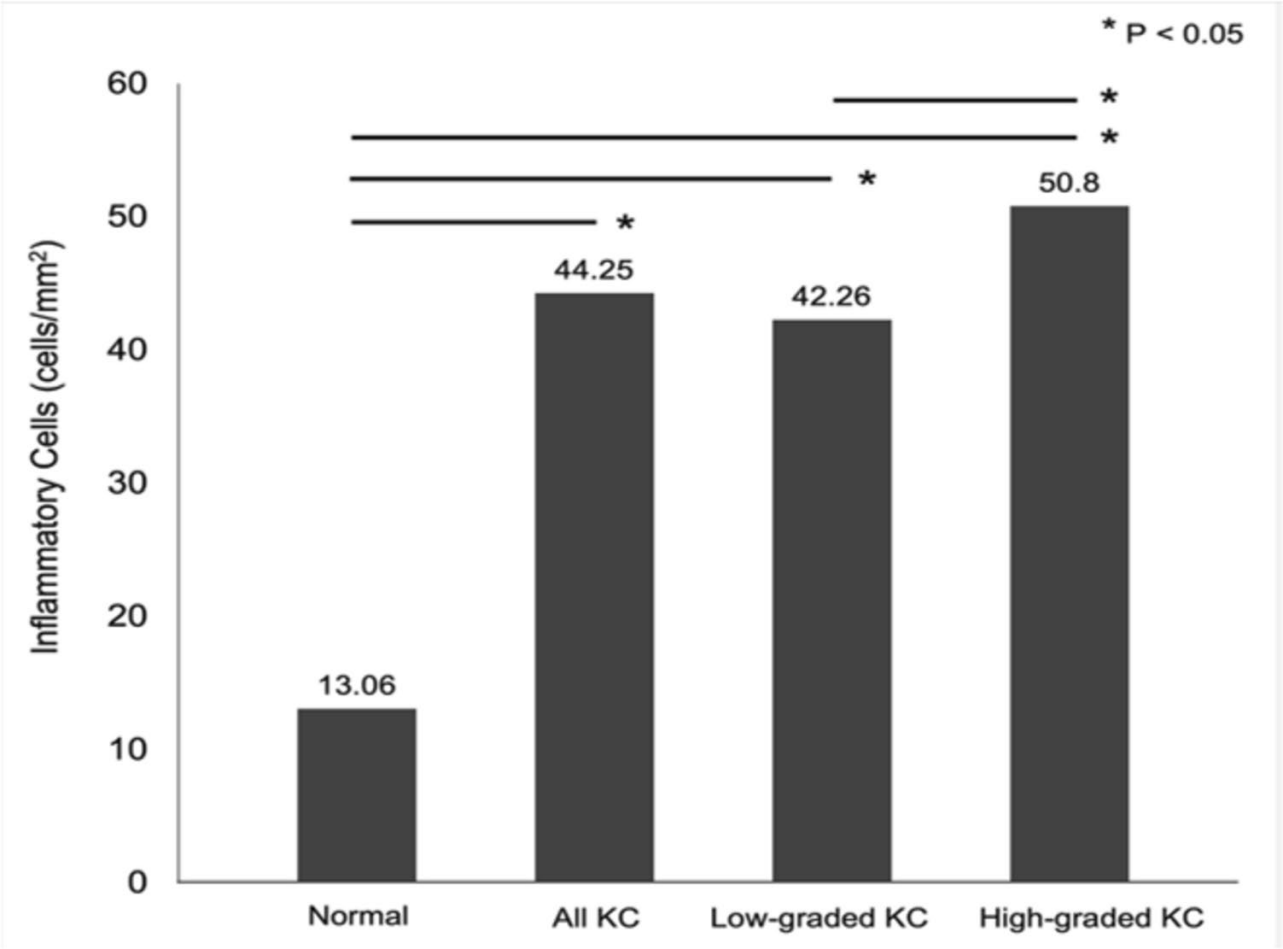
Comparison of inflammatory cells of patients with keratoconus (KC) and controls, detected by in vivo confocal microscopy

Regarding the primary outcome, the IC density was significantly higher in the KC group than in the controls (44.25 ± 7.01 vs. 13.06 ± 7.51 cells/mm^2^; *p* < 0.001). Moreover, patients with high-grade KC showed a significantly higher IC density than those with low-grade KC (42.26 ± 7.01 vs. 50.80 ± 8.43 cells/mm^2^; *p* < 0.001); Fig. 2). A rise in IC density was significantly associated with KC severity (*p* < 0.001, 95% CI [0.70, 0.95]).

As for the secondary outcomes, the subbasal nerve parameters detected in the central cornea of the patients with KC and the healthy subjects (nerve number, nerve density and nerve tortuosity) are detailed in Table 2. Although the number of main nerve trunks of the keratoconic eyes was not distinct from that of normal eyes (*p* = 0.107), the densities of the main nerve trunks, neve branches, and total nerves were significantly decreased in the keratoconic eyes relative to the controls (all *p* < 0.002). Furthermore, the reduction in total nerve density was more pronounced in high-grade KC than in low-grade KC (*p* = 0.001). However, we did not find a significant correlation between subbasal nerve density and the severity of KC (*p* = 0.25). Overall, the patients with KC had a slight loss of corneal sensitivity. The nerve tortuosity showed a bump in low-grade KC, but the overall keratoconic eyes were similar to controls.

**Table 2:** Subbasal nerve parameters analyzed by in vivo confocal microscopy between patients with keratoconus (KC) and controls

		KC		
Parameter	Normal	All	Low-grade	High-grade
Nerve number (number/mm^2^)				
Main nerve	28.46 ± 1.72	25.0 ± 1.31	27.68 ± 2.48	24.30 ± 1.51
Nerve Branch	63.83 ± 2.86	51.10 ± 5.09*	63.99 ± 6.28	47.76 ± 6.08*
Total nerve	92.29 ± 3.00	76.10 ± 5.48*	91.67 ± 6.25	72.07 ± 6.53*‡
Nerve density (mm/mm^2^)				
Main nerve	11.57 ± 0.64	9.30 ± 0.45*	10.16 ± 0.98	9.08 ± 0.51*
Nerve Branch	9.18 ± 0.38	7.24 ± 0.60*	9.57 ± 0.91	6.66 ± 0.69*^‡^
Total nerve	20.62 ± 0.72	16.54 ± 0.79*	19.63 ± 0.87	15.74 ± 0.91*^‡^
Nerve tortuosity (Grade 0–4)	2.28 ± 0.14	2.4 ± 0.10	2.71 ± 0.14	2.31 ± 0.11

Values are presented as mean ± standard error of mean, unless otherwise noted

*Statistical significance when *p* < 0.05 compared between study groups and control group

‡Statistical significance when *p* < 0.05 compared between study groups

Corneal nerve tortuosity was graded according to the classification of Oliviera-Soto et al

**Fig. 3:**
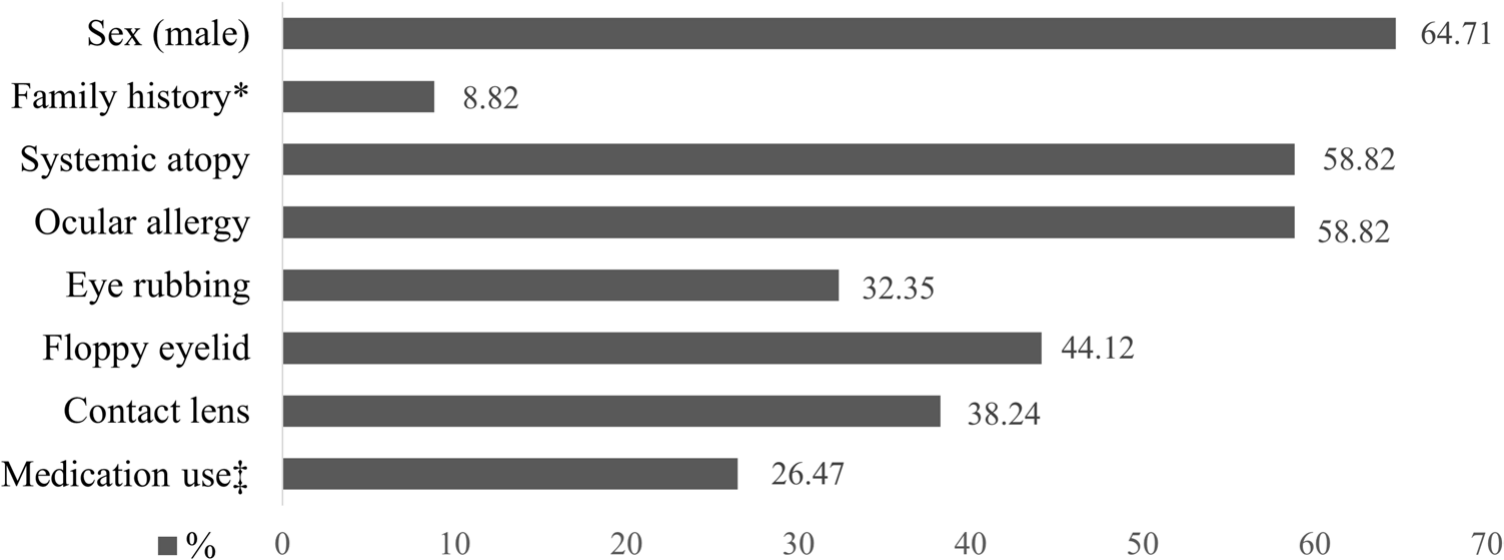
Keratoconus-related factors obtained from all patients.* Family history of keratoconus. Rigid gas permeable (RGP) lens use. ^‡^ Occasionally use oral antihistamine

The KC-related factors obtained from our patients are illustrated in Fig. 3. Systemic atopy and ocular allergies were found to be the most common (59%), followed by floppy eyelid (44%). None had used topical steroids or antihistamine eye drops.

There were no significant correlations between the KC-related factors and the number of corneal ICs, except the severity of KC (*p* < 0.001) Table 3. Although the number of subbasal nerves substantially declined in KC, we did not find a relationship between subbasal nerve density and the number of ICs (*p* = 0.835).

**Table 3:** Correlation between inflammatory cell density and Keratoconus-related factors

Associated factors	Coefficient	95%CI	(*p*-value)
Severity of KC	0.19	0.70 to 0.95	< 0.001*
Sex (male)	-0.15	-0.47 to 1.16	0.336
Family history of Keratoconus	0.10	-0.05 to 0.26	0.208
Systemic atopy	0.04	-0.18 to 0.26	0.702
Ocular allergy	-0.48	-0.28 to 0.18	0.690
Eye rubbing	1.30	-0.21 to 2.81	0.504
Floppy lid	0.11	-0.00 to 0.22	0.603
Contact lens Medication use	0.06 0.03	-0.17 to 0.30 -0.20 to 0.27	0.601 0.303

*Statistical significance when *p* < 0.05

## Discussion

This study explored corneal inflammation in KC by detecting ICs using laser IVCM. The investigations revealed a significant rise in ICs in keratoconic eyes relative to healthy corneas. An increase in the IC density of patients with KC is consistent with a decrease in the number of subbasal nerves. The alterations were more noticeable in high-grade KC than in low-grade KC. Our results confirm the presence of intracorneal inflammation and some degree of neurotrophic keratopathy in patients with KC.

In general, there are two main pathogeneses of corneal tissue degradation: degeneration and inflammation [34]. Theoretically, the etiology of progressive stromal loss in KC is presumed to arise from a degenerative process rather than an inflammatory response, as keratoconic eyes appear clinically uninflamed. However, several researchers have proposed that KC’s pathogenesis may involve chronic inflammatory pathways [35–38]. Elevated levels of diverse serum markers (eg. neutrophil/lymphocyte ratio, monocyte/high-density lipoprotein cholesterol ratio), indicating systemic immune responses, were also found in patients with KC [36–38].

Our study results concur with the inflammation hypothesis, as we found a significant rise in ICs in the subbasal layer of keratoconic corneas visualized by IVCM, relative to normal corneas. Moreover, the IC density was greater in high-grade KC than in low-grade KC. These findings demonstrate the existence of intracorneal inflammation in KC, with the degree of inflammation depending upon the severity of the disease. Mandathara et al. also reported concordant results, as they found a significantly high number of Langerhans cells (LCs) in their series of KC patients [39].

Typically, dendritiform LCs or ICs are rarely detected in the central cornea in the quiescent state. The migration of ICs from the periphery toward the central cornea indicates a trafficking pattern of ICs mediated by the immune response [21]. An influx of corneal ICs has been shown in various conditions, such as those found in infectious keratitis, corneal graft rejection, and vernal keratoconjunctivitis [20, 23, 29, 40, 41]. However, some indiscernible inflammation resulting from dry eye disease or prolonged contact lens wear can also induce a rise in IC numbers [20, 21, 42].

As we know, the pathophysiology of KC is multifactorial. Several KC-related factors are constituents and play a role in destroying corneal collagen. Ocular allergy and systemic atopy, often present in patients with KC, render high levels of pro-inflammatory cytokines in tears and serum [16–19, 36–38]. Floppy eyelid syndrome (FES), recognized as a common coexisting disorder of KC. Abnormalities in the tear film dynamics in FES generate a rise in MMP-9, which produces chronic ocular surface inflammation [43, 44]. Eye rubbing has also been documented as a cause of keratoconjunctivitis [45]. Based on the physiological role of the corneal epithelial basement membrane in controlling corneal homeostasis and wound healing, the inciting signal of epithelial trauma mediates an inflammatory cascade through the healing process. From scientific rationale mentioned above, we presumed that repetitive mechanical trauma from chronic eye rubbing in KC patients activates corneal inflammation.

Regarding contact lens use, studies have analyzed the effects of various contact lens types on the cornea [46, 47]. The prolonged use of contact lenses leads to mechanical corneal epithelial injury, which releases apoptotic cytokines [48]. Moreover, confocal microscopic studies have revealed a high corneal epithelial dendritic cell density in the central cornea of contact lens wearers. This finding suggests that a centripetal migration of ICs occurred in response to an inflammatory stimulus [42, 49].

In the current study, ocular allergies and systemic atopy were the most common KC-related factors, similar to reports in the literature [3, 4, 8]. One may think that the increased number of ICs detected in keratoconic eyes might be influenced by allergies. From our study, there was not associated between ocular allergies and IC density. Moreover, none of the KC-related factors were significantly correlated with the IC numbers detected by IVCM.

In actuality, any KC-related factor is a plausible cause of corneal inflammation. We were somewhat surprised not to find a correlation between KC-associated factors and IC density unlike the results from previous studies. However, those prior studies analyzed ocular surface inflammation in patients with specific conditions, and many of their cases exhibited similar features with clinically inflamed eyes, resulting in a positive correlation with ICs. In contrast, our KC patients had a variety of associated factors with different severities, and their eyes were clinically not inflamed. Therefore, it is possible that the relationship between each KC-related factor and the ICs may not have been detected. Nonetheless, most KC patients presented with composite factors. A cumulative level of inflammation could contribute to a significant increase in ICs, as we observed in confocal microscopy. In other word, an immune-inflammatory response may result from not just a single factor but several factors’ combined effect, yielding the distinctly high IC density of keratoconic corneas. Additionally, many patients with KC need to wear contact lenses to improve their vision. Thirty-eight percent of our patients had a history of RGP use. These patients were requested to refrain from wearing the lenses for at least a month before enrollment to obtain the decent corneal topographic evaluation. Nevertheless, no data indicate the duration needed to ensure that eyes are free of inflammation after discontinuing contact lenses.

The present study revealed that the density of ICs was greater in patients with high-grade KC than in those with low-grade KC, and the number of ICs showed a strong relationship with the severity of KC. It could be speculated that sustained exposure to inflammatory-precipitating factors could engender inflammation accumulating in the patients’ corneas, eventually progressing to an advanced KC stage.

Regarding corneal innervation, it has been widely demonstrated the structural changes of subbasal nerve plexus in KC. Many researchers have shown a reduced nerve length and density in confocal images, while some have observed increased nerve tortuosity in the cornea’s apex [50–53]. Our results also found subbasal nerve decline in both the number and density, with the decline correlating with a slight loss of corneal sensitivity. In our subgroup analysis, corneal nerves were significantly reduced in high-grade KC compared with low-grade KC. Several studies have shown that inflammatory cell infiltrates presented around corneal nerves were associated with various corneal diseases. Cruzat et al. demonstrated that perineural dendritic cells increase in parallel with a decrease of corneal nerves in patients with different types of infectious keratitis [54]. Hamrah et al. also revealed similar findings in herpes simplex virus (HSV) keratitis [26]. In addition, other research groups observed perineural infiltration corresponding to subbasal nerve changes in conditions such as aqueous tear deficiency dry eye [55] and vernal keratoconjunctivitis [32]. All this research evidently supports our findings that inflammatory cells located near subbasal nerves are implicated in corneal nerve alterations.

Interestingly, we observed an increase in nerve tortuosity only in low-grade KC. Kawabuchi et al. explained this morphological change in which nerve tortuosity was deemed to be a biomarker in representing nerve regeneration [56]. The researchers presumed that mild inflammation in the early stage of KC may cause minimal nerve damage; therefore, the remaining nerves can renew. As the inflammation persists and the disease progresses to a more advanced stage, the subsequent nerve destruction might be too severe to restore. Although significant nerve diminishment was detected in overall KC patients, we did not find an association between subbasal nerve numbers and IC density. We assume that the decreased nerve number in KC may derive from chronic subtle inflammation and also other factors, such as disorganized corneal stroma, which disallow proper nerve regeneration.

There are some limitations of this study: 1) We performed confocal analysis in both eyes of some patients if those eyes were eligible. Taking into account the same KC-related factors in both eyes of some patients, we modified the statistical analysis on a dependent-eye basis. However, a larger number of cases would yield more valid outcomes, and 2) we did not include patients with other inflammatory cornea and ocular surface diseases to compare with KC patients. The different confocal findings between KC and clinically inflamed eyes may offer new insights into the evolution and progression of KC.

## Conclusion

The current study demonstrates intracorneal inflammation in patients with KC detected by IVCM. An increase in ICs is not particularly associated with any KC-related factors, and a substantial rise in IC density is significantly correlated only with the severity of KC. The existing corneal inflammation in keratoconic eyes corresponds to a reduction in the subbasal nerve plexus. Proof of subclinical corneal inflammation and neurotrophic keratopathy in KC detected by IVCM may enlighten researchers about the inflammation-based pathogenesis and future remedies for KC.
